# Unilateral Cervical Polyneuropathies following Concurrent Bortezomib, Cetuximab, and Radiotherapy for Head and Neck Cancer

**DOI:** 10.1155/2016/2313714

**Published:** 2016-02-25

**Authors:** Alhasan Elghouche, Tom Shokri, Yewen Qin, Susannah Wargo, Deborah Citrin, Carter Van Waes

**Affiliations:** ^1^Indiana University School of Medicine, 1120 W. Michigan Street, Suite 200, Indianapolis, IN 46202, USA; ^2^Division of Otolaryngology, Penn State Milton S. Hershey Medical Center, 100 Campus Drive, Suite 400, Hershey, PA 17033, USA; ^3^King's College London School of Medicine, Strand, London WC2R 2LS, UK; ^4^Head and Neck Surgery Branch, National Institute on Deafness and Other Communication Disorders, 9000 Rockville Pike, Bethesda, MD 20892, USA; ^5^Radiation Oncology Branch, Center for Cancer Research, National Cancer Institute, 9000 Rockville Pike, Bethesda, MD 20892, USA

## Abstract

We report a constellation of cervical polyneuropathies in a patient treated with concurrent bortezomib, cetuximab, and cisplatin alongside intensity modulated radiotherapy for carcinoma of the tonsil with neck metastasis. The described deficits include brachial plexopathy, cervical sensory neuropathy, and oculosympathetic, recurrent laryngeal, and phrenic nerve palsies within the ipsilateral radiation field. Radiation neuropathy involving the brachial plexus is typically associated with treatment of breast or lung cancer; however, increased awareness of this entity in the context of investigational agents with potential neuropathic effects in head and neck cancer has recently emerged. With this report, we highlight radiation neuropathy in the setting of investigational therapy for head and neck cancer, particularly since these sequelae may present years after therapy and entail significant and often irreversible morbidity.

## 1. Introduction 

We report the case of a patient with head and neck cancer (HNC) who developed multiple distinct neuropathies following participation in a clinical trial in which she received concurrent bortezomib, cetuximab, cisplatin, and radiotherapy. The patient's neuropathies include brachial plexopathy, cervical allodynia, and oculosympathetic, recurrent laryngeal, and phrenic nerve palsies. This is the first case to describe such an array of deficits in the context of this particular treatment regimen.

Radiation-induced brachial plexopathy (RIBP) has long been recognized as a potential toxicity of radiation therapy in patients with carcinoma of the breast or lung. There is emerging recognition of the potential for RIBP in patients treated for HNC.

## 2. Case Presentation 

A 54-year-old female presented to her primary care provider with a one-month history of a lateral neck mass and concurrent pooling of salivary secretions. The patient reported current alcohol and tobacco usage with approximately 40-pack-year history. Her past medical history included chronic hepatitis C infection. Persistence of the neck mass despite antibiotic therapy prompted referral to an otolaryngologist whereupon the patient was noted to have an ulcerative lesion of the left palatine tonsil in addition to a left cervical mass measuring 2 × 3 cm. Fine needle aspiration and pathologic analysis of the cervical mass revealed squamous cell carcinoma (SCCa) positive for human papilloma virus. Two months after initial symptom onset, positron emission tomography/computed tomography revealed increased uptake in the left tonsillar region with a standardized uptake value (SUV) of 15.2 along with uptake in a left level II cervical lymph node with a SUV of 11.9. The patient was subsequently referred to the National Institutes of Health and diagnosed with a T1N2bM0, stage IVA SCCa of the oropharynx with primary site in the left palatine tonsil. In the time leading up to referral, the patient noted dysphagia, odynophagia, and intermittent otalgia. Physical examination was significant for an enlarged and erythematous left palatine tonsil that was firm to palpation in addition to left-sided jugulodigastric lymphadenopathy measuring 3.5 × 3 cm.

**Figure 1:**
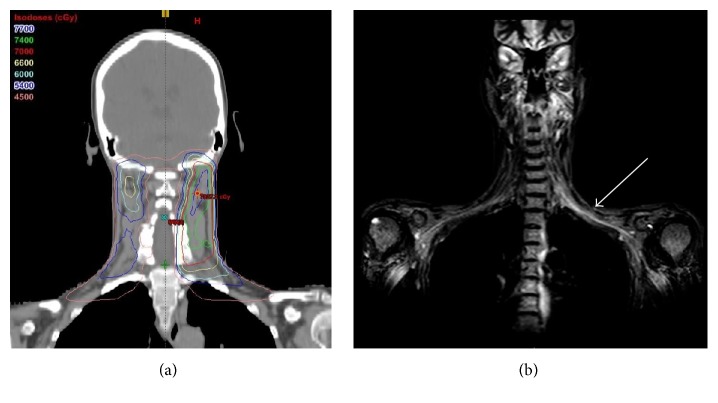
(a) Composite plan with coronal isodose distributions. (b) MRI of the brachial plexus, with T2 prolongation on the left (arrow). Cranial and brachial nerve roots emerge in isodose regions receiving 6600–7400 cGy.

Following direct laryngoscopy with biopsy of the left tonsil, the patient was enrolled in NCI-7893, an IRB approved Phase I dose escalation study of bortezomib, cetuximab, and Intensity Modulated Radiation Therapy (IMRT) [1]. Bortezomib (1.3 mg/m^2^) was given intravenously twice weekly on days 1, 4, 8, and 11, every 21 days. Bortezomib and cetuximab 400 mg/m^2^ were started one week before combining bortezomib and weekly cetuximab 250 mg/m^2^ with IMRT. However, during the first five weeks of treatment, the patient developed repeated infusion reactions to cetuximab despite reduced infusion rates and administration of antihistamine medication and steroids. No reduction in the left neck adenopathy was observed. It was therefore elected to remove the patient from the research protocol; her treatment regimen was transitioned to include a standard dose of cisplatin (100 mg/m^2^) during completion of the last 3 weeks of radiation therapy. The patient's planned radiation therapy of 70 Gray (Gy) delivered in daily 2 Gy fractions to the primary tumor and gross nodal disease was boosted to 74 Gy. A dose of 70 Gy was delivered in daily 2 Gy fractions to the high risk left neck, and a dose of 54 Gy was delivered to the lower left neck and low risk lymph nodes in the contralateral neck (Figure 1(a)).

Thirty months after completion of combined chemotherapy and radiotherapy, the patient developed intermittent paresthesia of the left hand and aching pain throughout the entire left upper extremity. On physical examination, the patient was found to have atrophy of the left forearm extensor musculature. Weakness was demonstrated with left shoulder abduction and left elbow, wrist, and finger extension. Subsequent physiatry assessment demonstrated a markedly reduced left-sided grip strength of 47 lbf compared to 58 lbf in the right hand. Electromyography (EMG) showed sensory nerve abnormalities in the left upper extremity. Magnetic resonance imaging (MRI) of the left brachial plexus revealed T2 prolongation within the C7 nerve root indicative of inflammation and/or edema (Figure 1(b)). The patient was diagnosed with radiation-induced brachial plexopathy (RIBP). Initially, the patient reported significant relief of pain with gabapentin and physical rehabilitation.

**Figure 2:**
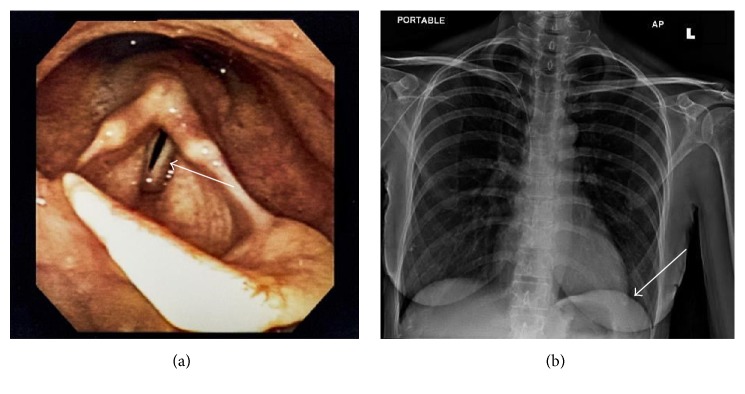
(a) Laryngoscopy photograph of the vocal folds, demonstrating left vocal fold paresis. (b) Portable chest radiograph with elevation of the left hemidiaphragm (arrow).

Three months after onset of upper extremity symptoms, the patient developed hoarseness and flexible laryngoscopy revealed left vocal fold paralysis and a pan-glottic gap with phonation (Figure 2(b)). The patient was also noted to have left-sided ptosis and miosis consistent with Horner's syndrome. Repeat MRI of the neck showed no evidence of recurrence and no mass lesions affecting the course of the left recurrent laryngeal nerve or carotid plexus. The patient's hoarseness was not responsive to corticosteroid administration and she underwent injection laryngoplasty with improvement in voice quality. The patient's condition remained stable until, nearly three years after completion of concurrent chemoradiotherapy, she began to experience severe allodynia described as a burning sensation extending from the left supraclavicular skin towards the left submandibular region and the superior aspect of the left shoulder. This pain was attributed to potential progressive cervical sensory neuropathies analogous to the patient's RIBP. Shortly thereafter, she also began to experience intermittent dyspnea both at rest and on exertion. Imaging of the chest suggesting elevation of the left hemidiaphragm and associated atelectasis (Figure 2(b)) was confirmed by fluoroscopy as consistent with left phrenic nerve palsy.

At the time of this writing, the patient's deficits were stable and/or partially responsive to oral narcotic analgesia and physical rehabilitation exercises.

## 3. Discussion 

The patient described above, following administration of three separate therapeutic agents alongside radiation therapy, experienced brachial plexopathy, cervical nerve irritation, and oculosympathetic, recurrent laryngeal, and phrenic nerve palsies in the ipsilateral radiation field.

The pathophysiology of radiation-induced neuropathy is attributed to fibrosis with entrapment of nerve fibers, ischemic injury from capillary disruption, demyelination, and direct axonal damage [2–4]. Onset of radiation neuropathy may occur months to decades after initial therapy and the deficits are typically irreversible [5].

RIBP is a delayed, progressive potential toxicity associated with irradiation of the neck and supraclavicular regions; clinical manifestations include pain, paresthesia, and weakness of the upper extremity [6]. Long recognized in the context of breast and lung cancers, increased awareness of RIBP in patients treated for HNC has emerged over the last five years [7]. A history of irradiation should increase clinical suspicion of the diagnosis, which may be confirmed via EMG and/or MRI. Distinction from recurrent malignancy or alternate primary is an important diagnostic consideration. A correlation has been shown between total radiation dose, fraction size, and the particular method by which the region is irradiated [8, 9]. However, the risk factors and associated radiation parameters remain poorly defined, particularly in patients with HNC in which higher doses of radiation are typically required [10–12].

Chen et al. prospectively screened 330 patients who completed radiation therapy for HNC using a standardized questionnaire in order to identify predictors of RIBP [7]. Their study showed that the use of concurrent chemotherapy increased the risk of developing neuropathy and that delivery of higher doses of radiation (defined as >70 Gy) to the brachial plexus was strongly associated with subsequent symptom development. These findings were consistent with prior recommendations by Emami et al. who postulated that treatments above 70 Gy drastically increased rates of injury [13]. However, there were a small proportion of patients in the study that developed symptoms attributable to RIBP below this dosing threshold indicating that the underlying mechanism of injury may be multifactorial, including an increased risk with concurrent chemotherapy highlighted in the above report.

Vocal fold paralysis (VFP) has also recently been cited as a delayed complication of radiotherapy for HNC [5]. A case series by Crawley and Sulica identified 10 patients with VFP attributable to radiation therapy for HNC with symptom onset ranging from one to 27 years following initial therapy. None of these patients experienced self-resolution of VFP. Injection augmentation was shown to be an effective management option in that case series and as affirmed by the response demonstrated in the above case report [5].

Phrenic nerve injury secondary to radiation therapy is rarely described, with only four previously reported cases. Regarding potential contributions from monoclonal antibody therapy, one case describes a patient with bilateral phrenic nerve injury secondary to ipilimumab [14]. Though the patient presented in this case report received a different monoclonal antibody, cetuximab, further study into potential adverse effects of treatment with monoclonal antibodies may be warranted. Cetuximab is an inhibitor of the epidermal growth factor receptor (EGFR). Two-thirds of patients enrolled in a Phase I study of the EGFR inhibitor gefitinib in combination with paclitaxel and radiotherapy were noted to develop severe oral dysesthesia [15]. In that study, the combination of an EGFR inhibitor appears to have exacerbated the potential neuropathic effects of paclitaxel with radiotherapy, since in previous trials of cetuximab in combination with radiotherapy for HNC, encompassing 227 patients, there were no reported instances of RIBP or other distinct nerve injuries as described in the present case report [14–16].

A common dose-limiting toxicity associated with bortezomib administration is painful sensory peripheral neuropathy [17, 18], and bortezomib may also have contributed to the cervical sensory and other neuropathies in combination with radiation described in this report. The effect may be dose dependent, as this was the only patient receiving 1.3 mg/m^2^ bortezomib, while other patients receiving lower doses in this and previous studies of bortezomib in combination with radiotherapy have not shown such limiting neurologic toxicities [1, 19]. The neuropathies described in this report cannot be attributed to acute bortezomib toxicity given that bortezomib-induced neuropathy typically manifests during the treatment course and has been shown to be reversible [19, 20]. Therefore, the sequelae exhibited in the above patient may indicate that the neurotoxicity of bortezomib potentiates, or is potentiated by, concurrent radiotherapy and/or EGFR inhibition. Cisplatin chemotherapy is also reported to cause peripheral sensory neuropathies [21–23]. A significant portion of the left neck received a dose of 70 Gy or higher due to hot spots in the composite treatment plans. The majority of the brachial plexus received less than 70 Gy; however, the patient's radiation exposure seems likely to have sensitized nearby nervous structures to the toxic effects of bortezomib, cetuximab, and cisplatin, given the polyneuropathic effects within the radiation field.

There exists the potential of an immune-mediated component to the described nerve injuries. One case report describes a chronic immune-mediated polyneuropathy five months following cetuximab administration for treatment of HNC [17]. EGFR inhibition has been shown to increase expression of proinflammatory genes including interleukin-6 (IL-6) [24]. Interestingly, bortezomib-induced neuropathy is associated with relative increases in the number of Th2 cells and IL-6 levels [25].

## 4. Conclusion 

The above case report demonstrates that concurrent administration of bortezomib in conjunction with cetuximab and radiotherapy is associated with an array of distinct cervical neuropathies. Radiation-induced nerve injuries, including RIBP, are an important and potentially underrecognized consideration in monitoring of patients treated for HNC. The clinician monitoring patients following chemoradiotherapy for HNC must be attuned to potential nerve injuries given the sometimes decades long latency to symptom onset, the significant impairment these toxicities entail, and their irreversible nature.
